# SASP PROTEIN ASSOCIATIONS WITH HUMAN AGING HIGHLIGHTS THE ROLE OF GDF15, CST3, AND IGFBP2

**DOI:** 10.1093/geroni/igac059.2929

**Published:** 2022-12-20

**Authors:** Daniel Evans, Danielle Young, Toshiko Tanaka, Nathan Basisty, Luigi Ferrucci, Judith Campisi, Birgit Schilling

**Affiliations:** California Pacific Medical Center, San Francisco, California, United States; California Pacific Medical Center, San Francisco, California, United States; NIA IRP, Baltimore, Maryland, United States; National Institute of Aging, Baltimore, Maryland, United States; National Institute on Aging, Baltimore, Maryland, United States; Buck Institute for Research on Aging, Novato, California, United States; Buck Institute for Research on Aging, Novato, California, United States

## Abstract

Cellular senescence is a hallmark of aging and can result in a Senescence-Associated Secretory Phenotype (SASP). SASP is characterized by an increased production of proinflammatory cytokines and damage to surrounding tissues. There is substantial evidence from non-human models demonstrating that SASP contributes to tissue dysfunction and pathogenic effects of aging. However, there are relatively fewer studies on the relationship between SASP and aging in humans. In this report, we determined the relationship between SASP circulating proteins and a wide range of aging-related clinical traits in two human cohort studies (BLSA and InChianti) using aptamer-based proteomics. Clinical traits examined were waist circumference, glucose levels, systolic and diastolic blood pressure, C-reactive protein, Interleukin-6, alkaline phosphatase, blood urea nitrogen, gait speed, grip strength, albumin, and red blood cell distribution width. In the meta-analysis of regression model results from both studies, 38 of the 77 SASP proteins examined were significantly associated with age. Of the 38 age-associated SASP, 26 were associated with one or more age-associated clinical traits. A cluster of three SASP proteins (GDF15, CST3, and IGFBP2) showed significant associations with inflammatory markers, renal function and hematological traits. A second cluster was significantly associated with grip strength and gait speed, two measures of physical function that are both strongly associated with mortality. Taken together, these results support the relevance of SASP proteins to human aging and point to specific protein components of the SASP that should be further examined for the potential to be biomarkers of human aging.

